# Effect of angiotensin receptor-neprilysin inhibitor on atrial electrical instability in atrial fibrillation

**DOI:** 10.3389/fcvm.2022.1048077

**Published:** 2022-12-08

**Authors:** Tianyu Zhu, Wenchao Zhang, Quan Yang, Ning Wang, Yuwei Fu, Yan Li, Guanliang Cheng, Liang Wang, Xian Zhang, Hongying Yao, Xinghe Sun, Yu Chen, Xiaohui Wu, Xuezhi Chen, Xiaohui Liu

**Affiliations:** ^1^Department of Cardiology, Peking University International Hospital, Beijing, China; ^2^Department of Ultrasound, Peking University International Hospital, Beijing, China

**Keywords:** ARNI, RFCA, atrial fibrillation, atrial electrical instability, structural remodeling

## Abstract

**Background and objective:**

Around 33.5 million patients suffered from atrial fibrillation (AF), causing complications and increasing mortality and disability rate. Upstream treatment for AF is getting more popular in clinical practice in recent years. The angiotensin receptor-neprilysin inhibitor (ARNI) is one of the potential treatment options. Our study aimed to investigate the effect of ARNI on atrial electrical instability and structural remodeling in AF.

**Methods:**

Our research consisted of two parts – a retrospective real-world clinical study and an animal experiment on calmness to verify the retrospective founding. In the retrospective study, we reviewed all patients (*n* = 110) who had undergone the first AF ablation from 1 August 2018 to 1 March 2022. Patients with ARNI (*n* = 36) or angiotensin II receptor antagonist (ARB) (*n* = 35) treatment were enrolled. Their clinical data, ultrasound cardiogram (UCG) and Holter parameters were collected before radiofrequency catheter ablation (RFCA) as baseline and at 24-week follow-up. Univariate and multivariate logistic regression analysis were performed. In the animal experiment, we established an AF model (*n* = 18) on canines by rapid atrial pacing. After the successful procedure of pacing, all the 15 alive beagles were equally and randomly assigned to three groups (*n* = 5 each): Control group, ARB group, and ARNI group. UCG was performed before the pacing as baseline. Physiological biopsy, UCG, and electrophysiological study (EPS) were performed at 8-week.

**Results:**

Clinical data showed that the atrial arrhythmia rate at 24-week was significantly lower in ARNI group compared to ARB group (*P* < 0.01), and ARNI was independently associated with a lower atrial arrhythmia rate (*P* < 0.05) at 24-week in multivariate regression logistic analysis. In the animal experiment, ARNI group had a higher atrial electrical stability score and a shorter AF duration in the EPS compared to Control and ARB group (*P* < 0.05). In the left atrium voltage mapping, ARNI group showed less low voltage and disordered zone compared to Control and ARB group. Compared to Control group, right atrium diameter (RAD), left ventricle end-diastolic volume index (LVEDVI), E/A, and E/E′ were lower in ARNI group (*P* < 0.05) at the 8-weeks follow-up, while left atrium ejection fraction (LAEF) and left ventricle ejection fraction (LVEF) were higher (*P* < 0.01). Compared to ARB group, LVEF was higher in ARNI group at the 8-week follow-up (*P* < 0.05). ARB and ARNI group had a lower ratio of fibrotic lesions in the left atrium tissues compared to Control group (*P* < 0.01), but no difference was found between the ARB and the ARNI group.

**Conclusion:**

ARNI could reduce atrial electrical instability in AF in comparison with ARB in both retrospective study and animal experiment.

## Introduction

Atrial fibrillation (AF) is defined as a tachyarrhythmia with uncoordinated atrial activation and ineffective atrial contraction (1). With 33.5 million patients worldwide, AF is the most common type of cardiac arrhythmia (2), leading to complication such as cerebral strokes and heart failure. It caused an increasing mortality and disability rate, leading to a higher health related economic burden (3).

More effective treatment for AF needs to be explored, especially for persistent AF. Most patients had a high risk of AF recurrence after cardioversion by classic anti-arrhythmic drugs (4). With the development of technology, radiofrequency catheter ablation (RFCA) was gradually becoming an effective treatment for AF, but the recurrence rate was still around 30% in the long-term (5). Considering the unsatisfactory results of classic antiarrhythmic drugs and RFCA (6), increasing attention was paid to the upstream treatment of AF, which could mechanically counter the atrial remodeling and therefore theoretically inhibit the initiation, maintenance, and progression of AF (7).

Angiotensin-converting enzyme inhibitor (ACEI) and angiotensin II receptor antagonist (ARB) were observed in experimental studies to be able to prevent the electrical and structural remodeling in AF (8, 9). In clinical practice, however, ACEI/ARB often played a role in primary prevention to reduce the incidence of new AF in patients with heart failure (10). Among patients already with AF, ACEI/ARB did not seem to be an effective treatment in secondary prevention in face of a recurrence rate with no statistical difference (11).

Valsartan/sacubitril, an angiotensin receptor-neprilysin inhibitor (ARNI), had gained increasing interest in the treatment of AF in recent years. In addition to the ARB effect with valsartan, ARNI also contains sacubitril, a neprilysin increasing the half-life of A-type natriuretic peptide (ANP) and B-type natriuretic peptide (BNP), which in turn results in a natriuretic, diuretic, vasodilatory, and antifibrotic effect (12). ARNI had been recognized as having superior effects to ACEI/ARB in the treatment of heart failure with the greatest mortality reduction (13). Several studies suggested that ARNI could decrease the atrial remodeling in heart failure and play a potential role in AF prevention (14). Dong et al. found ARNI was associated with a lower risk of AF recurrence compared to ACEI after RFCA in a propensity-matched cohort study (15). However, it was difficult to find whether it was the ARB or ARNI as a whole that resulted in a superior therapeutic effect to ACEI after the RFCA, given that ARB was one of the components of ARNI. There were few studies comparing the effects of ARNI and ARB on AF, especially from both clinical and experimental animal perspectives. Herein, we investigated the effects of ARNI in comparison with ARB on atrial electrical instability and structural remodeling in AF through retrospective clinical studies and canine animal experiments.

## Materials and methods

### Clinical data review

We retrospectively reviewed all patients who had undergone first AF ablation from 1 August 2018 to 1 March 2022 in the Heart Center of Peking University International Hospital. The data included the information below: (1) Basic information, including age, gender, height, weight, body mass index, and current smoker or drinker. (2) Medical history, including persistent atrial fibrillation (persistent AF), hypertension, diabetes mellitus, hyperlipidemia, congestive heart failure (CHF), myocardial infarction (MI), revascularization, peripheral vascular disease (PVD), stroke/transient ischemia attack (TIA), chronic obstructive pulmonary disease (COPD), chronic kidney disease (CKD), obstructive sleep apnea-hypopnea syndrome (OSAHS), and CHA2DS2-VASc score. (3) Perioperative medications that may affect the results. (4) Baseline clinical data at hospitalization before AF ablation, including heart rate (HR), systolic blood pressure (SBP), diastolic blood pressure (DBP), hemoglobin, serum creatinine, estimated glomerular filtration rate (eGFR), and serum potassium. (5) Ultrasound cardiogram (UCG) parameters at hospitalization before RFCA (baseline) and 24-week follow-up, including left atrium diameter (LAD), right atrium diameter (RAD), left ventricle end-diastolic diameter (LVEDD), right ventricle end-diastolic diameter (RVEDD), and left ventricle ejection fraction (LVEF). (6) Clinical data at 24-week follow-up, including AF recurrence or other atrial arrhythmia in Holter, all cause rehospitalization and all cause death.

The data was mainly collected by reviewing outpatient and inpatient medical history. For patients from provincial cities who were not able to be followed up in outpatient clinics, we used telephone follow-up to obtain information.

In this study, persistent AF was defined as AF lasting more than 7 days. AF early recurrence at 24-week follow-up was defined as AF sustained more than 30 s detected on Holter. Atrial arrhythmia at 24-week follow-up was defined as AF/flutter/tachycardia lasting longer than 3 beats or premature atrial contraction more than 1,000 beats per day on Holter.

**FIGURE 1:**
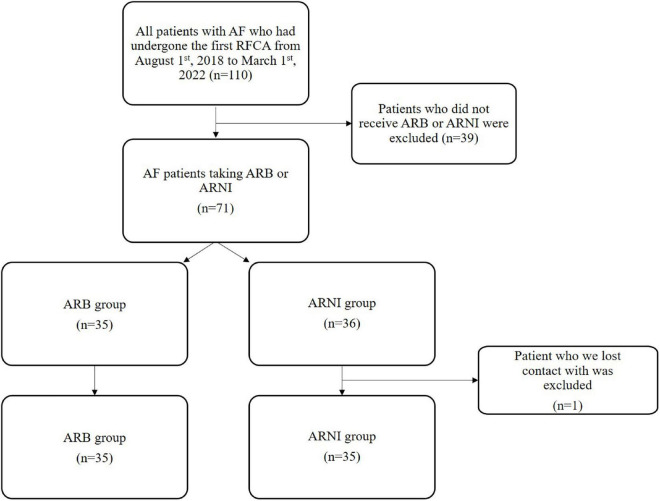
Flowchart of clinical retrospective study. AF, atrial fibrillation; RFCA, radio frequency catheter ablation.

Exclusion criteria: patients who did not take ARB or ARNI; patients we lost contact with. Patients treated with ARNI were selected to the ARNI group, and patients treated with ARB were selected to the ARB group (Figure 1).

The research was in compliance with the Declaration of Helsinki. The study protocol was approved by the Ethics Committee of Peking University International Hospital.

### Radiofrequency catheter ablation strategy

At baseline, electrocardiogram (ECG), Holter, and UCG were routinely performed for all patients. The AF was confirmed by ECG or Holter (AF on at least one ECG, or AF lasting more than 30 s on Holter). Anticoagulant such as Rivaroxaban or Dabigatran was routinely given to all patients before the RFCA for at least 3 weeks. Anti-arrhythmic drugs were discontinued for at least 5 half-lives before the RFCA. Transesophageal echocardiography was performed before the RFCA to rule out the embolism in left atrium (LA).

The ablation was performed under general anesthesia. After the successful punctures on bilateral femoral veins, the first dose of heparin was given (50 IU/kg). The decapolar catheter was placed in coronary sinus *via* the left femoral vein for electro-anatomical reference and stimulation. The LA access was established by an atrial septal puncture. After the successful atrial septal puncture, the second dose of heparin (50 IU/kg) was administered to maintain an activated clotting time at 250–350 s.

The Pentaray catheter (Pentaray Nav eco High-Density Mapping Catheter, Biosense Webster, CA, USA) was placed in the pulmonary veins (PVs) *via* the right femoral vein. The 3D mapping of LA was performed by CARTO system (CARTO 3, Biosense Webster, Yokneam, Israel). After the 3D mapping, the diagnostic/ablation deflectable tip catheter (THERMOCOOL SMARTTOUCH Catheter, Biosense Webster, Yokneam, Israel) was switched into the place for the subsequential ablations.

The PV antrum isolation was performed to block the atrial-PV bidirectional electrical conduction with a maximum power at 30–40 W, a maximum temperature at 43°C, an irrigation rate at 17–30 ml/min, and a minimum distance from the PV Ostia at 5 mm. If a non-PV trigger was present, such as LA posterior wall or superior vena cava, an additional isolation would be performed after the PV isolation. If there was an AF lasting more than 5 min after the isolations above, an additional ablation would be performed at the discretion of operator, such as LA linear (LA roofline and mitral isthmus line), cavotricuspid isthmus, and complex fractionated atrial electrogram (CFAE) ablation. An external electrical cardioversion would be performed to get the sinus rhythm if there was still an AF after all the ablations above.

Anticoagulants was continued after ablation. Anti-arrhythmic drugs such as Propafenone or Amiodarone were routinely administered within 3 months after RFCA if there was no contraindication. (Briefly: Propafenone for patients without heart failure and structural heart disease; Amiodarone for patients without hyperthyroidism, abnormal liver function and pulmonary fibrosis). Patients would be treated with only β-blocker because of the contraindications of both Propafenone and Amiodarone. No anti-arrhythmic drugs would be used if there was a bradycardia after RFCA, with the consent of operator.

### Establishing atrial fibrillation animal model

Eighteen male beagles (10–15 kg) were obtained from Nongnong Biotechnology Co., Ltd. (Beijing, China) (Supplementary material 1). ECG and UCG were performed to collect the baseline data. Canines with existing AF or low LVEF would be excluded. The canine model of AF was established in the same protocol as previously reported (16). All procedures were performed after sterile thoracotomy on the fifth intercostal space of the right chest under mechanical ventilation. The pacemaker was obtained from Xiamen Liqi Technology Co., LTD. (Fujian, China). The electrode was attached to the right atrium during the procedure. Then the pacemaker was inserted into a subcutaneous pocket on the back of canines. ECG was used to confirm the successful pacing. The pacemakers were initially turned off for the first week to give the canines a recovery time. Then we set an atrial rapid pacing at 500 beats per minute with 5 V square wave and 0.8 ms duration for 8 weeks (AOO mode). The canines were randomly divided into three groups as follows: Control group treated with placebo control (sausage without medication), ARB group treated with Valsartan (p.o. at a dose of 30 mg/kg/day, inserted into sausage; Novartis Pharma Schweiz AG, Switzerland), and ARNI group treated with Sacubitril/Valsartan (p.o. at a dose of 60 mg/kg/day, inserted into sausage; Novartis Pharma Schweiz AG, Switzerland). All medications were bought from the pharmacy of Peking University International Hospital. After 8 weeks of pacing, ECG was used to confirm the function of pacemaker. Canines that deceased and those with malfunction pacemaker would be excluded. The canines were anesthetized with 3% pentobarbital sodium (1 ml/kg) during the establishment of AF model, the echocardiography and the electrophysiological study (EPS).

### Electrophysiological study

After 8 weeks of continuous pacing, EPS was measured using DF-5A cardiac electrophysiological programmed stimulator (Dongfang Electronic Instrument Factory, Jiangsu, China) under the guidance of 3D electrophysiological navigation system (Biosense Webster, CA, USA). The electrode catheter was introduced into the right atrium *via* the right femoral vein. The LA voltage mapping was measured after the atrial septal puncture.

Atrial fibrillation was induced by eight S1S1 electrical stimuli at a pacing cycle length of 200, 170, and 150 ms, three times each in sequence. AF was defined as irregular atrial rates faster than 500 bpm associated with irregular AV conduction lasting more than 1,000 ms. The atrial electrical stability score was defined as the number of the first stimuli to induce AF, if there was no AF at the end of nine times electrical stimuli (three times in each pacing cycle length), the atrial electrical stability score would be 10 points at the final. The AF duration was defined as the time from the end of the stimuli to the first sinus P wave. If the AF was persistent, the AF duration was recorded as 60 s.

### Echocardiography

Ultrasound cardiogram parameters were measured at the baseline and 8 weeks after continuous pacing, using a GE video E9 ultrasonic diagnostic instrument, s5-1 probe, probe frequency 2.5–3.5 mhz. The experimental canines were placed in the horizontal position, connected with the electrocardiogram, and each parameter was measured and averaged after three cardiac cycles.

The 2D parameters are routinely measured. The left atrial anteroposterior diameter (LAD) and left ventricular end-diastolic diameter (LVEDD) were measured on the parasternal left ventricular long axis view. The RAD was measured on the four-chamber view. The left atrium volume (LAV) was measured by area length method in four-chamber and two-chamber view. The maximum left atrial volume (LAVmax) was measured when the mitral valve was about to open, the minimum left atrial volume (LAVmin) was measured at the peak of the R wave. Body surface area (BSA) = 10.1 × Weight^2/3^ × 10^–4^. Left atrium volume index (LAVI) = LAVmax/BSA. Left atrial ejection fraction (LVEF) = (LAVmax − LAVmin)/LAVmax × 100%. Simpson’s method was used to measure left ventricular end-diastolic volume (LVEDV) and left ventricular ejection fraction (LVEF). Left ventricular end-diastolic volume index (LVEDVI) = LVEDV/BSA. Pulsed Doppler was used to measure mitral orifice velocities E and A in the four-chamber view. Tissue Doppler was used to measure E′ on the left-ventricular side of the mitral annulus. The E/A and E/E′ were automatically calculated by the machine.

### Pathological staining

The experimental canines were euthanized after electrophysiological examination. The LA tissues were rapidly separated and preserved in formalin solution. The LA tissues were fixed in 10% formalin, dehydrated conventionally, embedded in paraffin, and sliced into 4 μm thick sections. The specimens were stained with Masson’s stain (Biotopped, Beijing, China) and examined under a light microscope (4 × 10 magnification). The ratio of fibrotic lesions was measured by ImageJ 1.8.0 (National Institutes of Health, Bethesda, MD, USA).

### Data and statistical analysis

Data are presented as mean ± standard deviation (SD) or mean ± standard error of the mean (SEM) for continuous variables and as frequency and percentages for nominal variables. Normally distributed continuous variables were compared using the Student’s *t*-test. The Pearson χ^2^ test was applied to all categorical variables. Logistic regression analysis was performed to evaluate the relation between ARNI and atrial arrhythmia at 24-week follow-up. A *P*-value of <0.05 was considered to be statistically significant. All statistical analyses were performed with SPSS v22.0 statistical software (SPSS, Chicago, IL, USA) and Prism 8.0 (GraphPad, San Diego, CA, USA).

## Results

### Baseline patient characteristics

A total of 110 patients’ data was retrospectively reviewed (Figure 1). Those who had neither ARNI or ARB were excluded (*n* = 39). The remaining 71 patients were enrolled for two groups: ARNI (*n* = 36) or ARB (*n* = 35). One patient we lost contact with in ARNI group was excluded. The basic information, the medical history and the clinical data at hospitalization before RFCA had no statistical difference between two groups (Table 1).

**TABLE 1:** Baseline clinical data.

Baseline data	ARNI	ARB	Differences between ARNI and ARB
**Basic information**			
Age	67.26 ± 11.97	65.2 ± 9.79	*P* = 0.434
Male gender	22/35	22/35	*P* > 0.999
Height (cm)	166.03 ± 8.48	166.89 ± 9.44	*P* = 0.691
Weight (kg)	70.46 ± 12.34	72.16 ± 13.47	*P* = 0.584
BMI (kg/m^2^)	25.47 ± 3.56	25.78 ± 3.38	*P* = 0.716
Current smoker	16/35	20/35	*P* = 0.339
Current drinker	15/35	18/35	*P* = 0.473
**Perioperative medications**			
Statin	18/35	20/35	*P* = 0.631
SGLT-2i	6/35	4/35	*P* = 0.495
Spironolactone	9/35	1/35	*P* = 0.006
**Preoperative anti-arrhythmic drugs**			
β-Blocker	21/35	17/35	*P* = 0.337
Amiodarone	2/35	3/35	*P* = 0.643
**Postoperative anti-arrhythmic drugs**			
β-Blocker	1/35	1/35	*P* > 0.999
Amiodarone	32/35	29/35	*P* = 0.284
Propafenone	2/35	5/35	*P* = 0.232
**Medical history**			
Persistent AF	16/35	10/35	*P* = 0.138
Hypertension	31/35	33/35	*P* = 0.393
Diabetes mellitus	16/35	19/35	*P* = 0.473
Hyperlipidemia	24/35	27/35	*P* = 0.420
CHF	16/35	14/35	*P* = 0.809
MI	3/35	2/35	*P* = 0.643
Revascularization	8/35	3/35	*P* = 0.101
PVD	4/35	2/35	*P* = 0.393
Stroke/TIA	14/35	11/35	*P* = 0.454
COPD	5/35	3/35	*P* = 0.452
CKD	4/35	3/35	*P* = 0.690
OSAHS	1/35	3/35	*P* = 0.303
CHA2DS2-VASc	3.54 ± 1.74	3.20 ± 1.53	*P* = 0.384
**Baseline clinical data**			
HR (beats per minute)	83.14 ± 20.34	80.03 ± 15.75	*P* = 0.476
SBP (mmHg)	131.49 ± 16.54	133.91 ± 15.66	*P* = 0.530
DBP (mmHg)	76.74 ± 15.88	73.77 ± 14.10	*P* = 0.411
Hemoglobin (g/L)	137.54 ± 18.48	139.43 ± 18.81	*P* = 0.674
Serum creatinine (μmol/L)	88.49 ± 38.97	76.66 ± 16.52	*P* = 0.103
eGFR (ml/min/1.73 m^2^)	77.11 ± 23.21	84.31 ± 14.54	*P* = 0.126
Serum potassium (mmol/L)	3.98 ± 0.30	4.11 ± 0.37	*P* = 0.108

Baseline characters had no statistical difference between two groups, except for usage of spironolactone.

BMI, body mass index; SGLT-2i, sodium-dependent glucose transporters inhibitor; AF, atrial fibrillation; CHF, congestive heart failure; MI, myocardial infarction; PVD, peripheral vascular; TIA, transient ischemia attack; COPD, chronic obstructive pulmonary disease; CKD, chronic kidney disease; OSAHS, obstructive sleep apnea-hypopnea syndrome; HR, heart rate; SBP, systolic blood pressure; DBP, diastolic blood pressure; eGFR, estimated glomerular filtration rate; RFCA, radiofrequency catheter ablation.

All medications that may affect AF outcomes were recorded (Table 1). Patients in these two groups were initially prescribed with ARB or ARNI, and did not change their treatment after the RFCA. All patients followed the anti-arrhythmic drugs strategy mentioned above, Amiodarone and Propafenone would be switched to the β-blocker 3 months after RFCA if there was no contraindication. The preoperative and postoperative anti-arrhythmic drugs were recorded in the Table 1. All these medications had no statistical difference between two groups, except for Spironolactone (ARB 1/35 vs. ARNI 9/35, *P* = 0.009).

At the baseline UCG data (Table 2), there were a significantly larger LVEDD (ARB 46.03 ± 5.37 mm vs. ARNI 50.09 ± 6.91 mm, *P* = 0.008) and a significantly lower LVEF (ARB 64.28 ± 1.34% vs. ARNI 56.05 ± 13.37%, *P* = 0.003) in ARNI group compared to ARB group. But LAD, RAD, and RVEDD shares no statistical difference between two groups.

**TABLE 2:** Ultrasound cardiogram parameters at baseline and 24-week follow-up.

UCG parameters	ARB			
	
	Baseline	24-week	Differences between baseline and 24-week	Change ratio
LAD (mm)	43.40 ± 6.05	41.74 ± 5.20	*P* = 0.105	−2.80 ± 11.29%
RAD (mm)	37.80 ± 6.18	34.91 ± 3.88	*P* = 0.012	−5.26 ± 12.45%
LVEDD (mm)	46.03 ± 5.37	47.11 ± 4.61	*P* = 0.164	+3.51 ± 11.40%
RVEDD (mm)	35.43 ± 4.08	32.03 ± 4.95	*P* = 0.001	−8.78 ± 14.39%
LVEF (%)	64.28 ± 1.34	65.21 ± 6.73	*P* = 0.647	+2.28 ± 13.78%

	**ARNI**			
	
	**Baseline**	**24-week**	**Differences between baseline and 24-week**	**Change ratio**

LAD (mm)	44.00 ± 6.83	40.13 ± 5.64	*P* < 0.001	−8.05 ± 10.16%
RAD (mm)	39.43 ± 6.93	34.83 ± 4.48	*P* < 0.001	−10.38 ± 11.67%
LVEDD (mm)	50.09 ± 6.91	48.70 ± 6.16	*P* = 0.064	−2.30 ± 8.43%
RVEDD (mm)	34.71 ± 5.29	31.71 ± 4.34	*P* = 0.010	−7.09 ± 16.15%
LVEF (%)	56.05 ± 13.37	59.87 ± 8.31	*P* = 0.021	+11.49 ± 24.65%

	**Differences between ARB and ARNI**		
	
	**Baseline**	**24-week**	**Change ratio**

LAD (mm)	*P* = 0.699	*P* = 0.223	*P* = 0.046
RAD (mm)	*P* = 0.303	*P* = 0.889	*P* = 0.083
LVEDD (mm)	*P* = 0.008	*P* = 0.232	*P* = 0.019
RVEDD (mm)	*P* = 0.529	*P* = 0.779	*P* = 0.648
LVEF (%)	*P* = 0.003	*P* = 0.005	*P* = 0.061

ARNI group had a significant larger LVEDD and lower LVEF at the baseline compared to ARB group. At the 24-week, the LVEF was still lower in ARNI group.

LAD and LVEDD change ratios were higher in ARNI group. LAD, left atrium diameter; RAD, right atrium diameter; LVEDD, left ventricle end-diastolic diameter; RVEDD, right ventricle end-diastolic diameter; LVEF, left ventricle ejection fraction.

Change ratio = (“24-week” − “baseline”)/“baseline” × 100%.

### Ultrasound cardiogram changes in patients

The UCG parameters were comparable between ARB and ARNI group (Table 2). At the 24-week follow-up, there was no difference in LAD, RAD, LVEDD, and RVEDD between the two groups. However, the LVEF was still significantly lower in ARNI group compared to ARB group (ARB 65.21 ± 6.73% vs. ARNI 59.87 ± 8.31%, *P* = 0.005). Compared to the baseline, both ARB group and ARNI group had a significantly lower RAD (ARB baseline 37.80 ± 6.18 mm vs. 24-week 34.91 ± 3.88 mm, *P* = 0.012; ARNI baseline 39.43 ± 6.93 mm vs. 24-week 34.83 ± 4.48 mm, *P* < 0.001) and RVEDD (ARB baseline 35.43 ± 4.08 mm vs. 24-week 32.03 ± 4.95 mm, *P* = 0.001; ARNI baseline 34.71 ± 5.29 mm vs. 24-week 31.71 ± 4.34 mm, *P* = 0.010) at the 24-week follow-up. Meanwhile, ARNI group also had significantly lower LAD (ARNI baseline 44.00 ± 6.83 mm vs. 24-week 40.13 ± 5.64 mm, *P* < 0.001) and larger LVEF (ARNI baseline 56.05 ± 13.37% vs. 24-week 59.87 ± 8.31%, *P* = 0.021) at the follow-up. Because of the differences in baseline UCG data between the two groups, it is possible that simply comparing the follow-up UCG data could not show the true picture. Therefore, we used the UCG change ratio [(“follow-up data” − “baseline data”)/“baseline data” × 100%] to describe the cardiac structural changes in these two groups. In ARNI group, the UCG change ratio showed a significant decrease in LAD (ARB −2.80 ± 11.29% vs. ARNI −8.05 ± 10.16%, *P* = 0.046) and LVEDD (ARB +3.51 ± 11.40% vs. ARNI −2.30 ± 8.43%, *P* = 0.019).

### Rhythm and major clinical events in different patient groups

The Holter was routinely measured at 24-week follow-up in all patients (Table 3). The atrial arrhythmias were significantly less in ARNI group (ARB 15/35 vs. ARNI 4/35, *P* = 0.003). Meanwhile, the AF early recurrence rate (ARB 4/35 vs. ARNI 2/35, *P* = 0.673) was lower in ARNI group with no statistical significance compared to ARB group. Besides, the rate of all cause rehospitalization and all cause death had no difference between the two groups (Table 3).

**TABLE 3:** Differences in rhythm and clinical events between groups.

	ARB	ARNI	Differences between ARB and ARNI
**Rhythm**			
AF early recurrence	4/35	2/35	*P* = 0.673
Atrial arrhythmia	15/35	4/35	*P* = 0.003
**Clinical event**			
All cause hospitalization	2/35	1/35	*P* = 0.555
All cause death	0/35	0/35	–

ARNI group had a significantly lower rate for atrial arrhythmia compared to ARB group.

There was no difference in all cause hospitalization and all cause death between groups.

AF, atrial fibrillation.

As there were differences at baseline data between ARB and ARNI groups such as LVEF, LVEDD, and preoperative medication, we performed a univariate logistic regression analysis for the presence of atrial arrhythmias at 24-week follow-up (Supplementary material 2). Factors with *P* < 0.2 in the univariate logistic regression analysis (ARNI and Statins in perioperative medications; Amiodarone in preoperative anti-arrhythmic drugs; persistent AF, diabetes mellitus, and stroke/TIA in medical history; SBP and serum potassium in baseline clinical data), factors that differed between the two groups at baseline (Spironolactone in perioperative medications; LVEF and LVEDD in baseline UCG data), and factors commonly considered to be associated with atrial electrical instability in clinic (LAD in baseline UCG data) were included in the multivariate logistic regression analysis (Table 4). The application of ARNI (*P* = 0.014, OR = 0.109) and Statins (*P* = 0.039, OR = 0.200) was independently associated with the atrial arrhythmia rate at 24-week follow-up.

**TABLE 4:** Multivariate logistic regression analysis for the presence of atrial arrhythmia at 24-week follow-up.

Multivariate logistic regression analysis for atrial arrhythmia rate			

Factors	*P*-value	OR	95% CI for OR
**Perioperative medications**			
ARNI	*P* = 0.014	0.109	0.019–0.635
Statin	*P* = 0.039	0.200	0.044–0.919
**Preoperative anti-arrhythmic drugs**			
Amiodarone	*P* = 0.174	8.341	0.392–177.276
**Medical history**			
Persistent AF	*P* = 0.417	2.127	0.344–13.161
Diabetes mellitus	*P* = 0.601	0.666	0.145–3.056
Stroke/TIA	*P* = 0.441	0.491	0.081–2.998
**Baseline clinical data**			
SBP (mmHg)	*P* = 0.287	1.027	0.978–1.079
Serum potassium (mmol/L)	*P* = 0.136	4.976	0.605–40.939
**Baseline UCG**			
LAD (mm)	*P* = 0.565	0.955	0.816–1.117
LVEDD (mm)	*P* = 0.670	1.032	0.894–1.190
LVEF (%)	*P* = 0.847	0.992	0.917–1.073

Factors with *P* < 0.2 in the univariate logistic regression analysis, factors that differed between the two groups at baseline and factors commonly considered to be associated with atrial electrical instability in clinic were included in the multivariate logistic regression analysis. ARNI and Statins were independently associated with the presence of atrial arrhythmia at 24-week follow-up (*P* < 0.05) in multivariate logistic regression analysis.

OR, odd ratio; CI, confidence interval; AF, atrial fibrillation; TIA, transient ischemia attack; SBP, systolic blood pressure; LAD, left atrium diameter; LVEDD, left ventricle end-diastolic diameter; LVEF, left ventricle ejection fraction.

### Baseline animal characteristics

A total of 18 beagles were entered into the experiment, and one was excluded at the beginning due to AF and low LVEF. The rest 17 canines underwent the right atrium pacing, and two of them deceased within 1 week after the procedure. A total of 15 canines were randomly divided into three groups. During 8 weeks of rapid pacing, one canine with malfunction pacemaker was excluded in ARNI group, and there was one deceased canine excluded in each of Control and ARB group. There were four canines in each group in the end (Supplementary material 1). All canines had similar length and weight in three groups (Supplementary material 3). All UCG data at baseline, including LAD, RAD, LAVI, LVEF, LVEDVI, LVEF, E/A, and E/E′, had no difference between all the groups (Table 5).

**TABLE 5:** Ultrasound cardiogram parameters in all animal groups.

UCG parameter	Control			
	
	Baseline	8-week follow-up	Differences between baseline and 8-week follow-up	
LAD (mm)	19.87 ± 0.80	24.39 ± 4.11	*P* = 0.096	
RAD (mm)	19.68 ± 0.92	23.96 ± 1.16	*P* = 0.048	
LAVI (ml/m^2^)	17.34 ± 0.89	22.76 ± 5.80	*P* = 0.206	
LAEF (%)	56.51 ± 3.11	34.63 ± 7.96	*P* = 0.016	
LVEDVI (ml/m^2^)	46.36 ± 2.73	54.29 ± 3.27	*P* = 0.115	
LVEF (%)	63.36 ± 4.38	55.47 ± 4.39	*P* < 0.001	
E/A	1.19 ± 0.05	1.89 ± 0.10	*P* = 0.018	
E/E′	8.27 ± 0.75	12.55 ± 0.78	*P* < 0.001	

	**ARB**			
	
	**Baseline**	**8-week follow-up**	**Differences between baseline and 8-week follow-up**	

LAD (mm)	19.98 ± 0.91	18.39 ± 2.18	*P* = 0.180	
RAD (mm)	18.77 ± 0.97	19.72 ± 2.88	*P* = 0.392	
LAVI (ml/m^2^)	14.20 ± 1.14	18.55 ± 2.87	*P* = 0.065	
LAEF (%)	56.06 ± 3.90	47.79 ± 5.69	*P* = 0.055	
LVEDVI (ml/m^2^)	46.30 ± 2.04	52.96 ± 15.72	*P* = 0.403	
LVEF (%)	64.57 ± 3.58	54.06 ± 6.60	*P* < 0.001	
E/A	1.13 ± 0.11	1.59 ± 0.20	*P* = 0.430	
E/E′	6.64 ± 1.17	9.31 ± 2.79	*P* = 0.012	

	**ARNI**			
	
	**Baseline**	**8-week follow-up**	**Differences between baseline and 8-week follow-up**	

LAD (mm)	21.33 ± 2.25	20.57 ± 5.18	*P* = 0.523	
RAD (mm)	19.12 ± 1.10	17.28 ± 4.29	*P* = 0.332	
LAVI (ml/m^2^)	14.71 ± 1.66	18.59 ± 3.85	*P* = 0.016	
LAEF (%)	57.05 ± 2.50	52.09 ± 2.97	*P* = 0.030	
LVEDVI (ml/m^2^)	43.73 ± 2.61	44.62 ± 6.30	*P* = 0.837	
LVEF (%)	64.26 ± 4.26	65.09 ± 2.56	*P* < 0.001	
E/A	1.12 ± 0.11	1.34 ± 0.13	*P* = 0.419	
E/E′	9.73 ± 1.66	7.63 ± 2.08	*P* = 0.008	

	**Differences at baseline between all groups**	**Differences at 8-week follow-up**		
		
		**Control vs. ARB**	**Control vs. ARNI**	**ARB vs. ARNI**

LAD (mm)	*P* = 0.745	*P* = 0.042	*P* = 0.291	*P* = 0.468
RAD (mm)	*P* = 0.810	*P* = 0.034	*P* = 0.024	*P* = 0.380
LAVI (ml/m^2^)	*P* = 0.227	*P* = 0.240	*P* = 0.275	*P* = 0.987
LAEF (%)	*P* = 0.977	*P* = 0.036	*P* = 0.006	*P* = 0.228
LVEDVI (ml/m^2^)	*P* = 0.703	*P* = 0.874	*P* = 0.034	*P* = 0.362
LVEF (%)	*P* = 0.977	*P* = 0.735	*P* = 0.009	*P* = 0.021
E/A	*P* = 0.849	*P* = 0.036	*P* = 0.001	*P* = 0.076
E/E′	*P* = 0.267	*P* = 0.066	*P* = 0.004	*P* = 0.369

There was no statistical difference at baseline between all groups. At 8-week follow-up, only LVEF was significantly larger in ARNI group compared to ARB group.

UCG, ultrasound cardiogram; LAD, left atrium diameter; RAD, right atrium diameter; LAVI, left atrium volume index; LAEF, left atrium ejection fraction; LVEDVI, left ventricle end-diastolic volume index; LVEF, left ventricle ejection fraction.

### Ultrasound cardiogram changes in animals

At 8-week UCG data (Table 5), LAD was lower in both ARB and ARNI groups compared to Control group, but was only statistically significant in ARB group (Control 24.39 ± 4.11 mm vs. ARB 18.39 ± 2.18 mm, *P* = 0.042). There was no statistical difference between ARB group and ARNI group. RAD was significantly lower in both ARB group and ARNI group compared to Control group (Control 23.96 ± 1.16 mm vs. ARB 19.72 ± 2.88 mm, *P* = 0.034; Control 23.96 ± 1.16 mm vs. ARNI 17.28 ± 4.29 mm, *P* = 0.024). However, there was still no significant difference between ARB and ARNI group. The similar characteristics were apparent in the left atrium ejection fraction (LAEF) and E/A. Compared to Control group, LAEF was significantly larger in both ARB and ARNI groups (Control 34.63 ± 7.96% vs. ARB 47.79 ± 5.69%, *P* = 0.036; Control 34.63 ± 7.96% vs. ARNI 52.09 ± 2.97%, *P* = 0.006), while E/A was significantly lower at the same time (Control 1.89 ± 0.10 vs. ARB 1.59 ± 0.20, *P* = 0.036; Control 1.89 ± 0.10 vs. ARNI 1.34 ± 0.13, *P* = 0.076), with no statistical difference between ARB and ARNI group. LVEF had no difference between Control and ARB group, but was significantly larger in ARNI group (Control 55.47 ± 4.39% vs. ARNI 65.09 ± 2.56%, *P* = 0.009; ARB 54.06 ± 6.60% vs. ARNI 65.09 ± 2.56%, *P* = 0.021). LVEDVI (Control 54.29 ± 3.27 ml/m^2^ vs. ARNI 44.62 ± 6.30 ml/m^2^, *P* = 0.034) and E/E′ (Control 12.55 ± 0.78 vs. ARNI 7.63 ± 2.08, *P* = 0.004) were also significantly lower in ARNI group compared to Control group, but had no difference between ARB and ARNI group.

### Histological changes in animals

**FIGURE 2:**
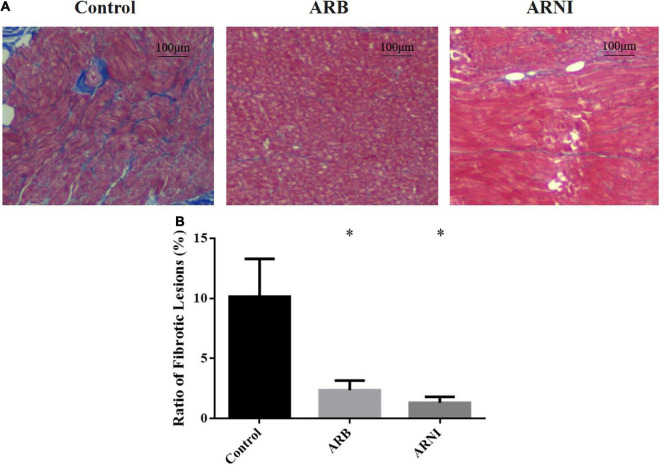
Pathological staining of left atrium. **(A)** Masson staining (4 × 10 magnification) renders myocardial cells red and collagen blue. The left atrium tissue in Control group had more fibrotic area compared to the other groups. **(B)** Ratio of fibrotic lesions in left atrium. *P < 0.05 compared to the Control group.

In the pathological staining results (Figure 2A), Control group had more fibrotic lesions compared to ARB and ARNI group in LA tissues. The ratio of fibrotic lesions (Figure 2B) was lower in both ARB (Control 10.15 ± 1.57% vs. ARB 2.36 ± 0.40%, *P* = 0.003) and ARNI group (Control 10.15 ± 1.57% vs. ARNI 1.31 ± 0.25%, *P* = 0.001) compared to Control group. There was no statistical difference between ARB and ARNI group (ARB 2.36 ± 0.40% vs. ARNI 1.31 ± 0.25%, *P* = 0.066), although ARNI group had a trend of a lower ratio of fibrotic lesions.

### Electrophysiological study in animals

**FIGURE 3:**
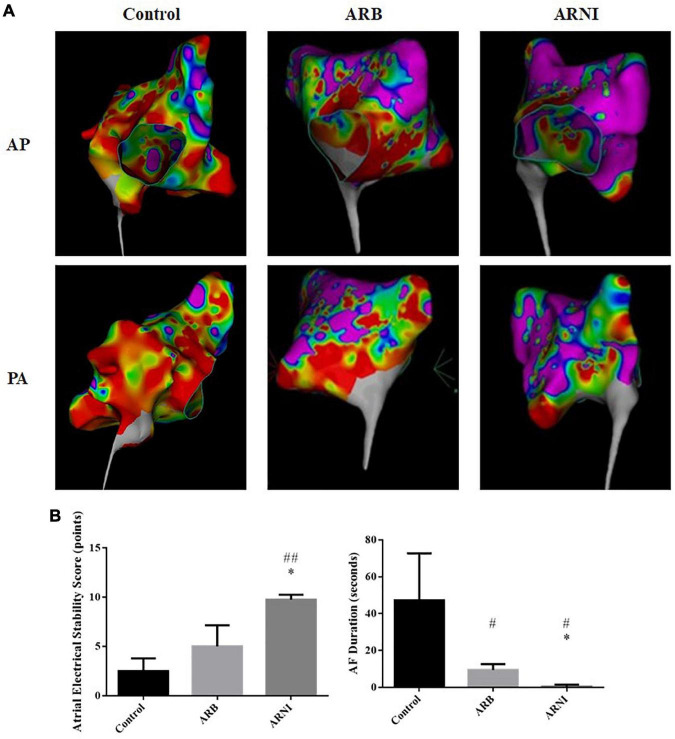
Electrophysiological study data. **(A)** LA voltage mapping in sinus rhythm in chest PA and AP views. Red area represents a low LA voltage zone with bipolar peak-to-peak electrogram voltage <0.50 mv. Purple area represents bipolar peak-to-peak electrogram voltage >3.0 mv. ARNI group had less low voltage and disordered voltage zone in left atrium. **(B)** Data after electrical stimuli. ARNI group had a higher atrial electrical stability score and a shorter AF duration. ^#^*P* < 0.05 compared to Control group; ^##^*P* < 0.01 compared to the Control group; *P < 0.01 compared to ARB group. AF, atrial fibrillation; LA, left atrium; AP, anteroposterior; PA, posteroanterior.

In the LA voltage mapping (Figure 3A), LA in ARNI group had less low voltage zone with red color and less disordered voltage zone with heterogeneous colors compared to ARB and Control groups, indicating a more stable atrial electrical activity. In the EPS, all canines did not have spontaneous AF. After the electrical stimuli, ARNI group showed a significantly higher atrial electrical stability score (Control 2.5 ± 1.29 pts vs. ARNI 9.75 ± 0.50 pts, *P* < 0.001; ARB 5 ± 2.16 pts vs. ARNI 9.75 ± 0.50 pts, *P* = 0.005) and shorter AF duration (Control 47.25 ± 25.5 s vs. ARNI 0.50 ± 1.00 s, *P* = 0.011; ARB 9.50 ± 3.11 s vs. ARNI 0.50 ± 1.00 s, *P* = 0.001) compared to ARB group and Control group (Figure 3B).

## Discussion

Our study investigated the effect of ARNI compared to ARB on atrial electrical instability and structural remodeling. We found the application of ARNI was independently associated with a lower atrial electrical instability in both retrospective study and canine AF model, while the difference of structural remodeling was only significant in the 24-week follow-up clinical study but not in the 8-week animal study.

In recent years, RFCA was gradually becoming the first-choice treatment for AF because of its advantages over classic antiarrhythmic drug therapy in all-cause mortality, HF hospitalization, LVEF, and quality of life (17). We therefore chose patients who had undergone RFCA, rather than those treated with classic antiarrhythmic drugs, for our retrospective study.

In our study, patients in ARNI group had worse structural parameters at baseline, including a larger left ventricle and a lower ejection fraction, which usually leads to a higher AF recurrence rate and a higher atrial electrical instability (18). However, patients in ARNI group had a lower rate of atrial arrhythmia at 24-week follow-up compared to ARB group in our study, although the AF early recurrence rate had no statistical difference. Besides, the application of ARNI was an independent protective factor for the presence of atrial arrhythmia at the 24-week follow-up in multivariate analysis. It was reasonable to speculate that ARNI performed better than ARB on reducing atrial electrical instability in patients with AF, as the worse cardiac structure at baseline and the lower atrial electrical instability at follow-up were found in ARNI group. Furthermore, ARNI group also had a higher reduction ratio in LAD and LVEDD, suggesting the advantage of ARNI group over ARB group on cardiac structural remodeling.

Atrial structural remodeling, especially atrial fibrosis occurs with disease progression in AF, which is an important cause of atrial electrical instability (19, 20). The role of ACEI/ARB in the inhibition of fibrosis in heart failure was well recognized. In patients with heart failure, ACEI/ARB could reduce the incidence of AF and other types of arrhythmias (21). However, there was a lack of strong evidence of ACEI/ARB in the secondary prevention of AF (22). The application of ARNI in patients with heart failure could lead to better clinical outcome than ACEI/ARB (23, 24). ARNI could attenuate cardiac remodeling after MI through a superior inhibition on fibrosis and hypertrophy than ARB (25). Russo et al. found that ARNI could reduce atrial and ventricular arrhythmias in patients with reduced ejection fraction and implantable cardiac defibrillator, indicating the potential effect in primary prevention of AF in patients with heart failure (26). Yang et al. found that LAD and RAD were lower in ARNI group patients compared to ARB group at the 24-week follow-up after AF ablation, indicating that ARNI is superior to ARB in attenuating atrial structural remodeling in RFCA-treated AF patients (27). Wang et al. found patients in ARNI group had a lower AF recurrence rate at 1-year follow-up after persistent AF catheter ablation compared to ARB group (28).

To sum up, there were scarce clinical studies on ARNI application in AF treatment as previous studies had mainly demonstrated a unilateral benefit of ARNI in electrical or structural terms. Our study demonstrated an extra clinical benefit of ARNI on both structural remodeling and atrial electrical instability in AF patients.

To further understand the mechanism by which ARNI benefits AF patients, we performed the animal experiment on canines to get data of UCG, EPS, and pathological staining. Our study found that ARNI group had a higher atrial electrical stability score and a shorter AF duration compared to ARB and Control group, indicating a superior protective effect on atrial electrical instability. The LA voltage mapping confirmed this conclusion, with less LA low voltage and disordered voltage zone in ARNI group.

Li et al. found that ARNI could ameliorate the atrial fibrosis and elevate the AF inducibility in rabbit AF models (29). Li et al. found ARNI could inhibit angiotensin II induced atrial fibrosis and therefore had a better effect than ARB in decreasing the AF susceptibility in rat AF model (30). Our animal experiment on canines was consistent with these studies regarding its influence on atrial electrical instability in AF. Nevertheless, previous animal studies had suggested that ARNI could reduce the atrial electrical instability through inhibition of atrial structural remodeling, but our animal experiments on canines had shown a different result.

In ARNI group, although RAD and LVEDVI was significantly lower at 8-week follow-up compared to Control group, all structural parameters such as LAD, RAD, LAVI, and LVEDVI had no statistical difference compared to ARB group. The pathological staining results were consistent with these UCG results, with a lower ratio of fibrotic lesions in ARNI group compared to Control group but no statistical difference when comparing to ARB group. In clinical practice, the additional effect of ARNI to reverse cardiac structural remodeling may take 6–12 months to occur (31). Therefore, it is possible that the difference in structural remodeling between ARNI and ARB group was not found in this 8-week canine experiments. Obviously, ARNI could attenuate cardiac structural remodeling in AF, but its superior effect over ARB on atrial electrical instability was not only contributed by the inhibition of cardiac structural remodeling in our experimental study. Similar results were reported in a study with left atrial appendage closure rabbit model (32), Cheng et al. found that ARNI could suppress the atrial arrhythmogenicity by increasing the level of ANP, even though the fibrosis between groups had no statistical difference.

Besides the inhibition of cardiac structural remodeling, the improvement in cardiac function by ARNI was thought to have potential anti-arrhythmic effects (14). De Vecchis et al. found that ARNI group had a higher increase of the peak atrial longitudinal strain and a lower risk of AF recurrence compared to the conventional therapy group in patients with heart failure and at least one episode of AF in the history (33). Suo et al. found that ARNI could lead to a superior improvement in left atrial function than ARB in both AF patients and pressure overload mouse model (34).

In our animal experiment, at 8-week follow-up, E/A and E/E′ were significantly lower, while LAEF was significantly higher in ARNI group compared to Control group. Besides, LVEF was significantly higher in ARNI group in compared with both ARB and Control group. Also, there was a trend towards higher LAEF in ARNI group compared to ARB group. These results indicated a better atrial and ventricular function in ARNI group. In the absence of significant difference between ARNI and ARB group in cardiac structural remodeling, it was a plausible explanation that ARNI could reduce atrial electrical instability by improving cardiac function. This phenomenon demonstrated that the effect of reducing atrial electrical instability after ARNI administration may occur rapidly with the protection of cardiac function, rather than until after the cardiac structural remodeling has taken place.

Calcium handling system of atrial myocytes was also considered to be related to AF (35). Extracellular calcium (Ca^2+^) enters myoplasm *via* activation of voltage-gated L-type Ca^2+^ channel (LTCC) and sodium-calcium exchanger (NCX) during the cardiac action potential and excitation-contraction coupling. This Ca^2+^ entry activates the ryanodine receptor 2 (RyR2) channel, triggering a larger amount of Ca^2+^ release from the sarcoplasmic reticulum (SR), resulting in myofilament activation. For relaxation, cytosolic Ca^2+^ was uptake back to SR *via* sarco-endoplasmic reticulum calcium ATPase 2a (SERCA2a) and excluded from cytoplasm to extracellular space *via* NCX and plasma membrane calcium ATPase (PMCA), allowing Ca^2+^ to dissociate from the myofilaments (36). High level of SR Ca^2+^ leak triggered by increased RyR2, together with upregulated NCX, could contribute to the pathogenesis of AF (37). Acute upregulation of SERCA2a by doxycycline in the setting of hyperactive RyR2 exacerbates dysregulated myocyte Ca^2+^ handling and arrhythmogenesis in both ventricular and atrial myocardium in rodent model (38). However, overexpression of SERCA2a could suppress ERP shortening and AF induced by rapid pacing atrium in rabbit model (39). The stress kinase c-Jun N-terminal kinase 2 (JNK2), a key factor to activate calmodulin-dependent protein kinase II (CaMKII), could stimulate the SERCA2a activity by regulating CaMKII-dependent arrhythmic SR Ca^2+^ leak and a CaMKII-independent uptake, which in turn exacerbates atrial arrhythmogenicity (40). Previous study had shown that ARNI could reduce the atrial arrhythmogenicity by reversing the remodeling of RyR2 channels and NCX1 channel (32). The Jun N-terminal kinases (JNKs) could be inhibited by ARNI in mice with diabetic cardiomyopathy (41), which may possibly result in a different activity of SERCA2a and CaMKII. The pathways above could be the other potential mechanisms for ARNI to affect atrial electrical instability in AF. This could to be further explored in the future.

### Limitations

For patients with AF and hypertension, our Heart Center prefer to use ACEI/ARB/ARNI to attenuate the cardiac remodeling in clinical practice. Therefore, we could not find a sizable control group with AF and hypertension but not taking the above medications for this retrospective real-world study. In clinical practice, ARNI and spironolactone were recommended to add in patients with definite ventricular enlargement and reduced ejection fraction (42), so the differences in LVEF, LVEDD, and spironolactone usage rate at baseline between groups were unavoidable in this retrospective study. The AF recurrence rate at 24-week had no statistical difference between groups, probably due to the short period of follow-up and the small sample size. More studies with longer follow-up period and bigger sample size are still needed to elucidate the role of ARNI in AF.

## Conclusion

In summary, the application of ARNI was independently associated with a lower incidence of atrial arrhythmia, which may result from reducing atrial electrical instability, when comparing to ARB for treating AF patients after their first RFCA. Therefore, we believe that ARNI could be a rational treatment in secondary prevention of AF.

## Data availability statement

The raw data supporting the conclusions of this article will be made available by the authors, without undue reservation.

## Ethics statement

The studies involving human participants were reviewed and approved by the Ethics Committee of Peking University International Hospital. Written informed consent for participation was not required for this study in accordance with the national legislation and the institutional requirements. The animal study was reviewed and approved by the Peking University Institutional Review Board.

## Author contributions

TZ: methodology, investigation, data curation, formal analysis, and writing—original draft. WZ: conceptualization, investigation, and resources. QY: investigation, data curation, and validation. NW: investigation and validation. YF and YL: investigation and data curation. GC, LW, XZ, HY, XS, YC, and XW: investigation. XC: methodology, investigation, data curation, resources, and writing—review and editing. XL: supervising, project administration, and foundation acquisition. All authors contributed to the article and approved the submitted version.
