# Supplementary material for: Effect of angiotensin receptor-neprilysin inhibitor on atrial electrical instability in atrial fibrillation
Source: Front Cardiovasc Med. 2022 Dec 8;9:1048077. doi: 10.3389/fcvm.2022.1048077 (PMC9772445; doi:10.3389/fcvm.2022.1048077)
Supplement: Supplementary file 1 [file Data_Sheet_1.docx]

**Supplement 1. Protocol of animal experiment.**


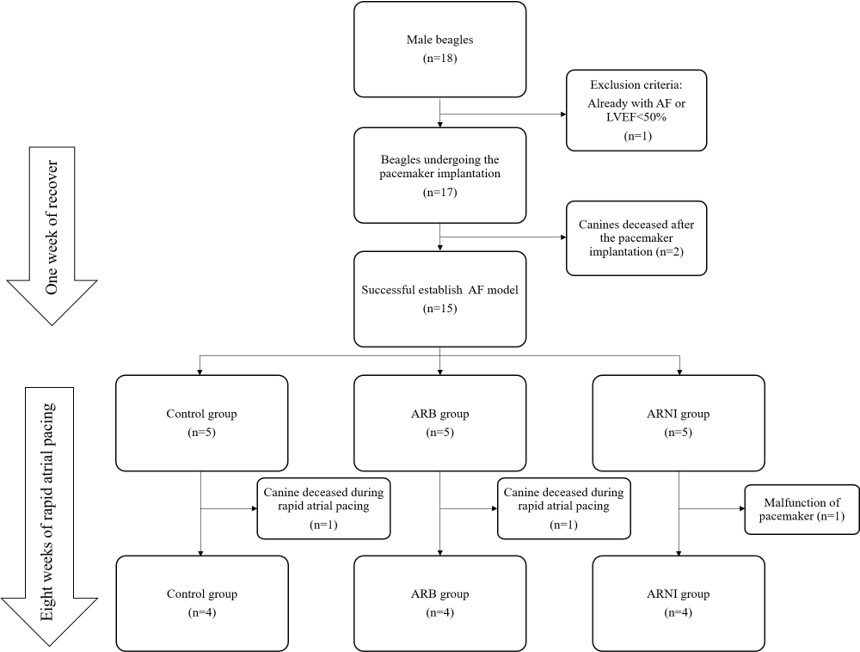


AF, atrial fibrillation; LVEF, left ventricle ejection fraction.

**Supplement 2. Univariate logistic regression analysis for the presence of atrial arrhythmia at 24-week follow-up.**

| **Factors** | ***P*-value** | **OR** | **95% CI for OR** |
| --- | --- | --- | --- |
| **Basic information** |  |  |  |
| Age | *P*=0.271 | 1.029 | 0.978-1.084 |
| Male Gender | *P*=0.557 | 1.398 | 0.457-4.279 |
| BMI (kg/m^2^) | *P*=0.268 | 0.912 | 0.775-1.073 |
| Current Smoker | *P*=0.902 | 1.068 | 0.372-3.067 |
| Current Drinker | *P*=0.982 | 1.012 | 0.352-2.908 |
| **Perioperative Medications** |  |  |  |
| ARNI | *P*=0.005 | 0.172 | 0.050-0.593 |
| Statin | *P*=0.078 | 0.376 | 0.127-1.118 |
| SGLT-2i | *P*=0.216 | 0.259 | 0.031-2.200 |
| Spironolactone | *P*=0.586 | 0.632 | 0.122-3.286 |
| **Preoperative Anti-arrhythmic Drugs** | | | |
| β-blocker | *P*=0.865 | 0.913 | 0.317-2.624 |
| Amiodarone | *P*=0.111 | 4.594 | 0.704-29.988 |
| **Postoperative Anti-arrhythmic Drugs** | | | |
| β-blocker | *P*=0.510 | 2.588 | 0.153-43.760 |
| Amiodarone | *P*=0.656 | 0.711 | 0.159-3.183 |
| Propafenone | *P*=0.929 | 1.082 | 0.192-6.115 |
| **Medical History** |  |  |  |
| Persistent AF | *P*=0.975 | 0.982 | 0.330-2.218 |
| Hypertension | *P*=0.722 | 0.723 | 0.121-4.314 |
| Diabetes Mellitus | *P*=0.183 | 0.479 | 0.162-1.415 |
| Hyperlipidemia | *P*=0.924 | 1.059 | 0.322-3.490 |
| CHF | *P*=0.938 | 0.959 | 0.330-2.784 |
| MI | *P*=0.508 | 1.882 | 0.289-12.247 |
| Revascularization | *P*=0.472 | 0.549 | 0.107-2.809 |
| PVD | *P*=0.722 | 1.382 | 0.232-8.244 |
| Stroke/TIA | *P*=0.126 | 0.381 | 0.111-1.311 |
| COPD | *P*=0.885 | 0.882 | 0.162-4.805 |
| CKD | *P*=0.433 | 0.417 | 0.047-3.710 |
| OSAHS | *P*=0.921 | 0.889 | 0.087-9.109 |
| CHA2DS2-VASc | *P*=0.319 | 0.842 | 0.600-1.181 |
| **Baseline Clinical Data** |  |  |  |
| HR (beats per minute) | *P*=0.915 | 0.998 | 0.969-1.028 |
| SBP (mmHg) | *P*=0.120 | 1.029 | 0.993-1.066 |
| DBP (mmHg) | *P*=0.471 | 1.013 | 0.978-1.049 |
| Hemoglobin (g/L) | *P*=0.956 | 1.001 | 0.973-1.030 |
| eGFR (mL/min/1.73m^2^) | *P*=0.292 | 1.016 | 0.986-1.047 |
| Serum Potassium (mmol/L) | *P*=0.071 | 4.715 | 0.876-25.375 |
| **Baseline UCG** |  |  |  |
| LAD (mm) | *P*=0.889 | 0.994 | 0.915-1.080 |
| RAD (mm) | *P*=0.891 | 1.006 | 0.928-1.090 |
| LVEDD (mm) | *P*=0.961 | 0.998 | 0.919-1.083 |
| RVEDD (mm) | *P*=0.346 | 1.055 | 0.944-1.178 |
| LVEF (%) | *P*=0.476 | 1.018 | 0.970-1.068 |

Only ARNI was associated with atrial arrhythmia rate at 24-week follow-up (*P*<0.05) in univariate logistic regression analysis.

OR, odd ratio; CI, confidence interval; BMI, body mass index; AF, atrial fibrillation; CHF, congestive heart failure; MI, myocardial infarction; PVD, peripheral vascular; TIA, transient ischemia attack; COPD, chronic obstructive pulmonary disease; CKD, chronic kidney disease; OSAHS, obstructive sleep apnea-hypopnea syndrome; HR, heart rate; SBP, systolic blood pressure; DBP, diastolic blood pressure; eGFR, estimated glomerular filtration rate; RFCA, radiofrequency catheter ablation; LAD, left atrium diameters; RAD, right atrium diameters; LVEDD, left ventricle end-diastolic diameters; RVEDD, right ventricle end-diastolic diameters; LVEF, left ventricle ejection fraction.

**Supplement 3. Baseline canine characteristics.**

| Baseline Data | Control | ARB | ARNI | Differences Between Groups |
| --- | --- | --- | --- | --- |
| Length | 80.25±6.60 | 80.25±3.30 | 81.0±6.68 | *P*=0.978 |
| Weight | 12.25±2.06 | 12.25±1.66 | 13.63±1.44 | *P*=0.465 |

There was no statistical difference between all groups.
